# Analysis of the frontal recess pneumatization pattern in patients with chronic frontal sinusopathy

**DOI:** 10.1016/j.bjorl.2025.101593

**Published:** 2025-03-26

**Authors:** Krystal Calmeto Negri, Rogério Hamerschmidt, Cassio Iwamoto, Carolina Rodrigues Laranjeira Vilar

**Affiliations:** a Universidade Federal do Paraná (UFPR), Curitiba, PR, Brazil; b Universidade Federal do Paraná (UFPR), Serviço de Otorrinolaringologia, Curitiba, PR, Brazil; c Hospital de Clínicas da Universidade Federal do Paraná (UFPR), Curitiba, PR, Brazil

**Keywords:** Frontal sinus, Frontal sinusitis, Computed tomography, Frontoethmoidal cells

## Abstract

**Objectives:**

To analyze the pneumatization pattern of the frontal recess using CT scans and to determine the prevalence of frontoethmoidal cells and their possible correlation with the development of sinusopathy.

**Methods:**

By means of a retrospective, analytical and cross-sectional study, 300 CT scans of patients with clinical suspicion of chronic rhinosinusitis were examined, separately on the right and left sides, totaling a sample of 600 paranasal sinuses, with regard to the presence of frontal cells, the presence of blockage or veiling of the recess and frontal sinus.

**Results:**

Frontoethmoidal cells were present in 85.8% of cases; the most frequent cells were supra bulla cells, in 43.8%, and the least frequent were supraorbital ethmoid cells, in 11% of cases. There was a 35% prevalence of supra-Agger cells, 15.8% of supra-Agger frontal cells, 20.2% of supra bulla frontal cells and 12.3% of frontal septal cells. A significant relationship was found between the presence of supra-Agger frontal cells and supra bulla frontal cells and the development of sinusopathy.

**Conclusion:**

The supra-Agger frontal cells and supra bulla frontal cells, when present in the frontal recess, predispose to the development of frontal sinusopathy. Therefore, preoperative tomographic analysis allows a three-dimensional anatomical understanding of the recess and frontal sinus based on determining the pneumatization pattern of this region.

**Level of evidence:**

Level 3.

## Highlights

• The supra-Agger frontal and supra bulla frontal cells predispose sinusopathy.
• Supra bulla frontal cells are independent factors for the outcome.
• Preoperative tomographic analysis provides greater safety to the surgery.
• Tomographic analysis allows anatomical understanding of the recess and frontal sinus.

## Introduction

Chronic rhinosinusitis is defined as an inflammatory disease of the nasosinusal mucosa that persists for at least twelve weeks. It has a multifactorial cause often associated with allergies, environmental, systemic and genetic factors.^1^ However, current knowledge of its pathogenesis does not allow us to clearly define a single inflammatory pathway that explains the entire process, from the initial injury to the structural changes in the sinonasal tissue. Adequate ventilation of the paranasal sinuses is necessary to maintain a healthy mucosa. Obstruction of the drainage ostia can predispose to the development of chronic rhinosinusitis.^2^ Mucociliary transport also plays an important role in this pathogenesis, as it is responsible for draining the mucus produced within the sinus into the nasal cavity.

**Fig. 1:**
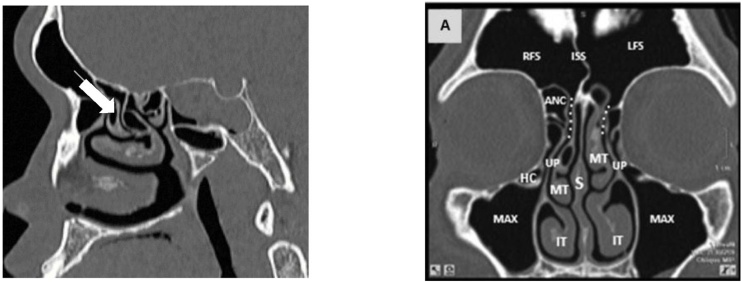
Normal anatomy of frontal recess: sagittal and coronal CT. Normal frontal recess anatomy. Sagittal CT shows the right frontal recess (white arrow). Computed tomography coronal section depicting the anatomy of the frontal sinus drainage pathway and surrounding structures. ANC, Agger Nasi Cell; RFS, Right Frontal Sinus; White dotted line frontal sinus drainage pathway; ISS, Intersinus Septum; IT, Inferior Turbinate; LFS, Left Frontal Sinus; Max, Maxillary sinus; MT, Middle Turbinate; S, Nasal Septum; HC, Haller Cell; UP, Uncinate Process.

Endoscopic sinus surgery has become one of the most commonly performed surgical procedures among otorhinolaryngologists. This therapeutic modality has been used to treat a wide variety of sinus diseases, one of the main indications being chronic rhinosinusitis. Surgical manipulation of the nasofrontal region remains a challenge even for experienced surgeons due to the complexity and anatomical variability of the three-dimensional spaces called the ethmoid infundibulum and frontal recess (Fig. 1).

Other factors that contribute to greater surgical difficulty in this region are: the need for an angled endoscopic access, since this area is located behind the frontal process of the maxilla (frontal beak), and the delicate anatomical relationship with neighboring noble structures, which carries a potential risk of injury to the lateral lamella of the lamina cribrosa, the lamina papyracea and the anterior ethmoidal artery, a vascular structure present in this location.^3^

**Fig. 2:**
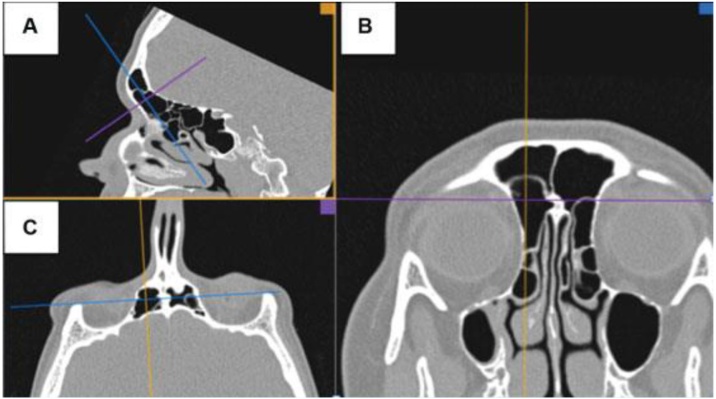
Multiplanar tomographic reconstruction. Multiplanar reconstruction of Computed Tomography (CT) scan using OsiriX DICOM viewer. (A) Parasagittal CT section, (B) Oblique section for true coronal plane, (C) Oblique section for true axial plane.

A perfect understanding of this anatomy allows for the development of operative techniques, safer surgical procedures and a higher success rate through complete dissection of the frontal recess.^4^ The gold standard exam for diagnosis and preoperative planning is computed tomography of the paranasal sinuses, as it provides high-resolution images less than 1 mm thick, allowing for a three-dimensional reconstruction of the local anatomy (Fig. 2). In addition, it is an efficient way of identifying anatomical variations in this region, which are not necessarily pathological, but which can add complexity to the anatomy of the lateral nasal wall, contributing to the occurrence or persistence of chronic inflammatory processes, as well as making it difficult to perform surgical procedures.

**Fig. 3:**
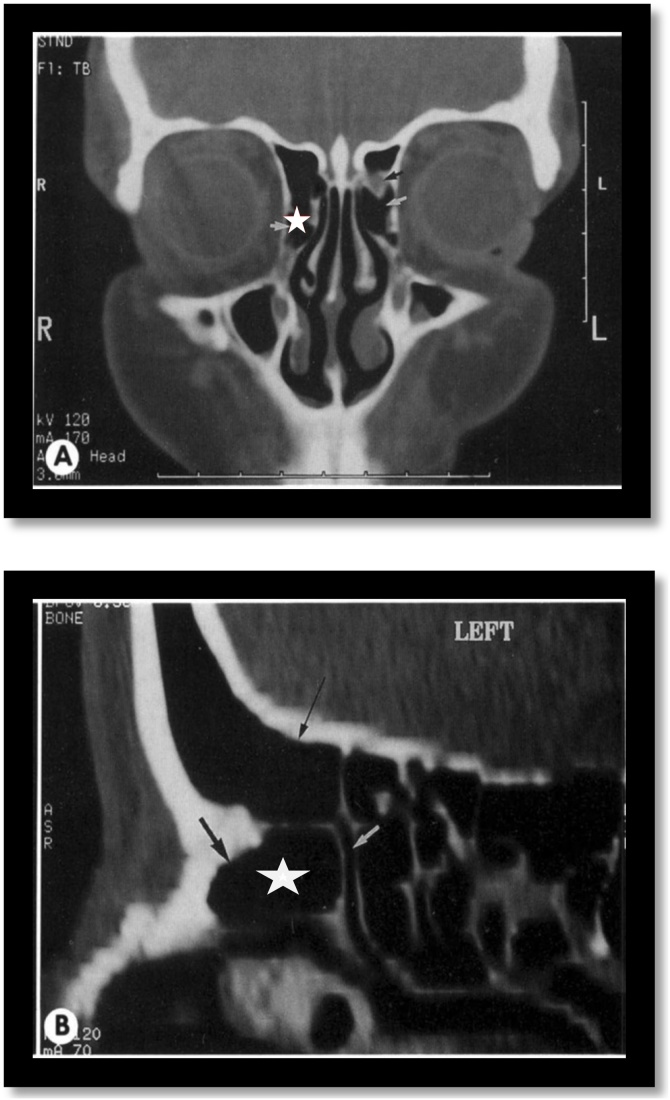
Agger nasi cell. Coronal and Sagittal computed tomography sections demonstrate Agger nasi cell (white star).

The frontal recess region is a narrow, inverted funnel-shaped space that is widely variable. The most commonly described anatomical variations involve the superior insertion of the unciform process; the pneumatization of the Agger nasi (Fig. 3); the bulla and the other cells present in the frontoethmoidal region. The posterior wall of the agger nasi and the anterior face of the ethmoidal bulla form, respectively, the anterior and posterior limits of the frontal recess and can potentially narrow this region according to their degree of pneumatization and the consequent spatial relationship.^5^, ^6^

**Fig. 4:**
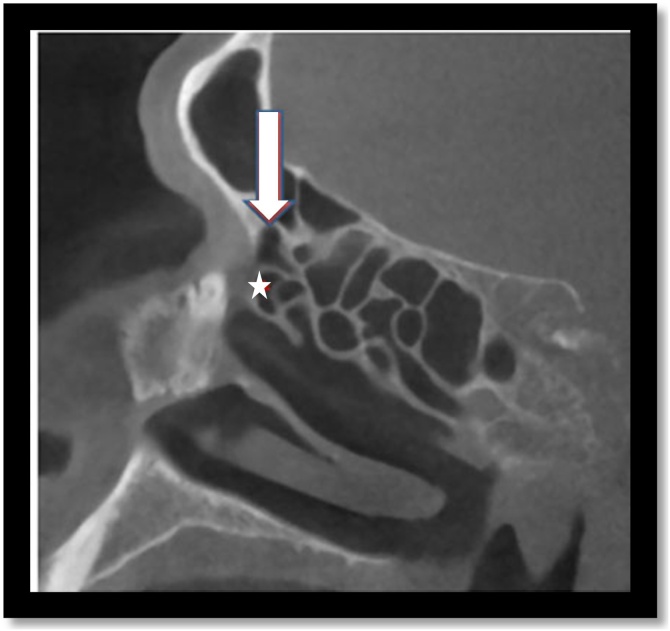
Supra-Agger cell. Sagittal computed tomography section demonstrates an example of Agger nasi cell (white star) and supra-Agger cell (white arrow).

**Fig. 5:**
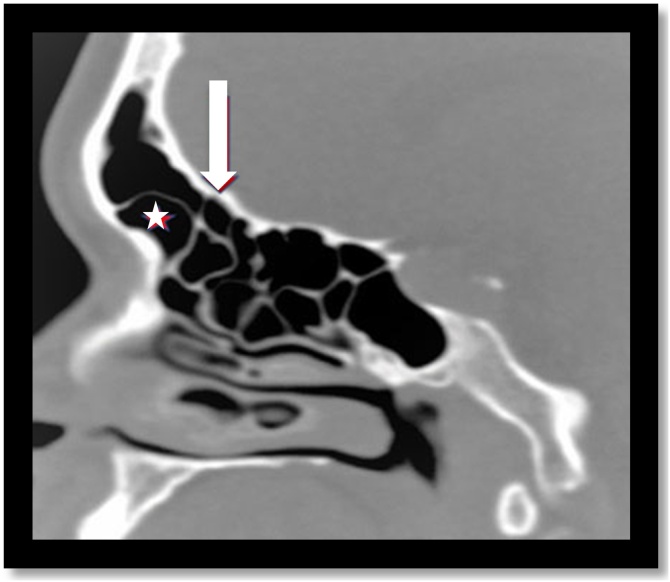
Supra-Agger frontal cell. Sagittal computed tomography section demonstrates an example of supra-Agger frontal cell (star) and supra bulla frontal cell (white arrow).

**Fig. 6:**
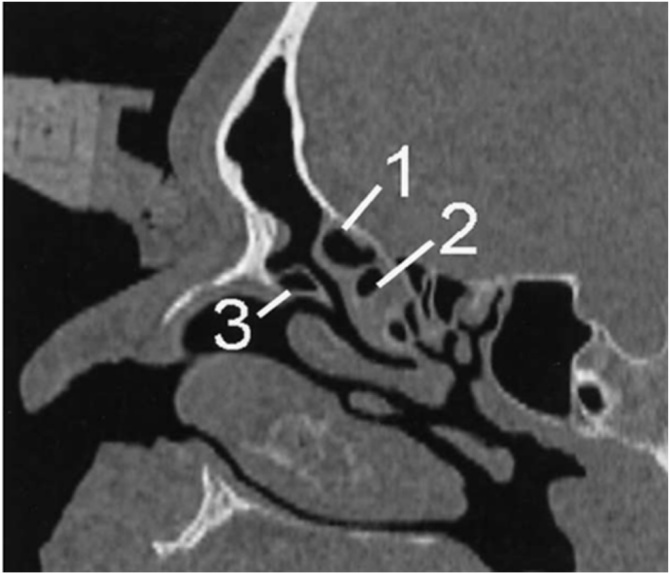
Bulla ethmoidalis. Sagittal computed tomography section demonstrates supra bulla cell (1) that is above the bulla ethmoidalis (2). An Agger nasi cell (3) is also shown.

**Fig. 7:**
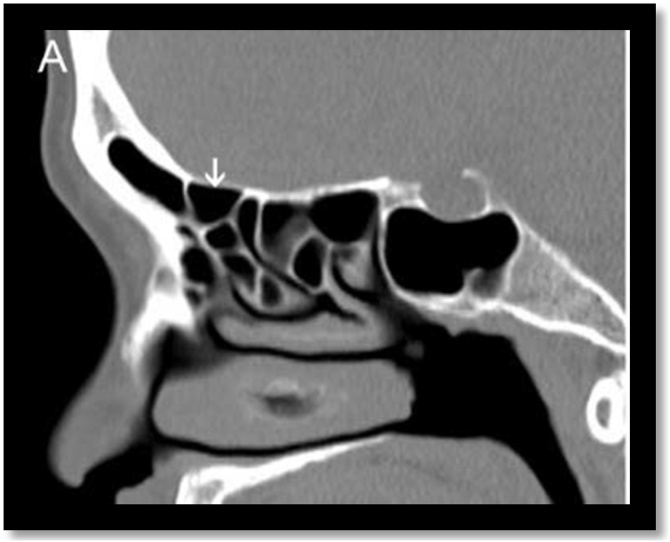
Supra bulla cell. Sagittal computed tomography section demonstrates an example of supra bulla cell (white arrow).

**Fig. 8:**
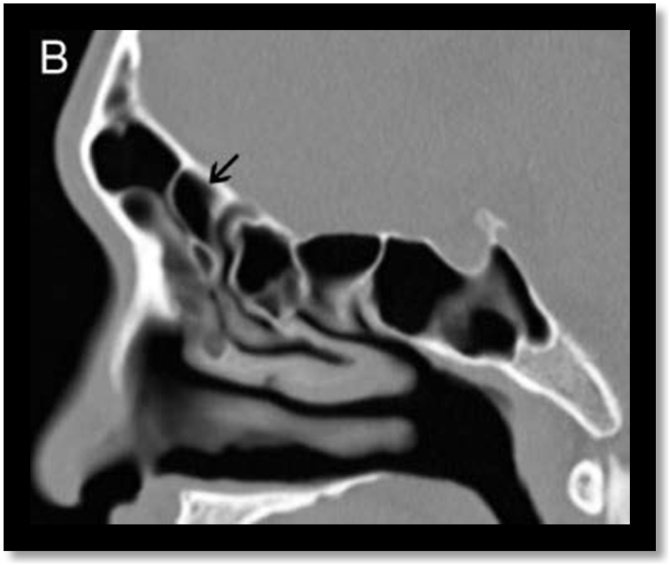
Supra bulla frontal cell. Sagittal computed tomography section demonstrates a supra bulla frontal cell (black arrow).

**Fig. 9:**
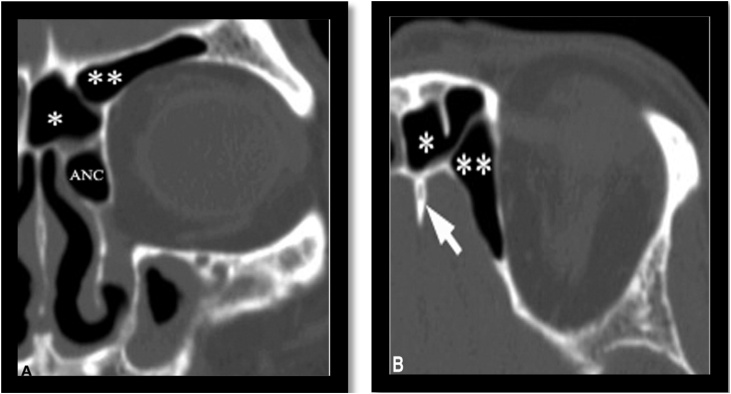
Supraorbital ethmoid cell. (A) Coronal plane CT images illustrates two cells in the left frontal region. The most lateral one is the supraorbital ethmoid cell (**) and the medial one is the frontal sinus merged with the IFSC (*). (B) Axial plane CT images illustrates three cells in the left frontal region. The most lateral one is the supraorbital ethmoid cell (**), the most medial one is the frontal septal cell (*), and in the middle is the frontal sinus. The crista galli is indicated (arrow). (ANC, Agger Nasi).

**Fig. 10:**
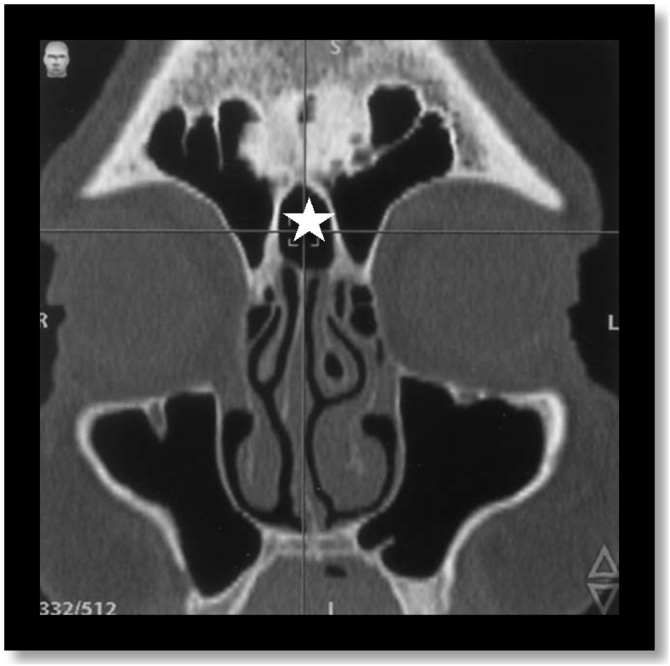
Frontal septal cell. Coronal plane CT images illustrates the frontal septal cell as a central compartment within the frontal bone (white star).

Other types of cells can be present in this region, called frontoethmoidal cells, and include: supra-Agger cells and supraorbital ethmoid cells, supra bulla frontal cells, supra bulla cells and frontal septal cells (Fig. 4, Fig. 5, Fig. 6, Fig. 7, Fig. 8, Fig. 9, Fig. 10). The frontal cells were first described by Schaeffer in 1916. In 1994, Bent e Kuhn created a classification of four types of frontal cells, according to their development above the Agger nasi; subsequently, some other classifications were proposed, the most recent being established in 2016. Theoretically, this set of cells could invade the frontal recess region, thus influencing the drainage of the frontal sinus.^5^, ^7^

The frontal recess region is a key area in the pathogenesis of frontal sinus disease^3^ and, since the presence of frontal cells may be associated with thickening of the frontal sinus mucosa and the development of frontal sinusitis, this study aims to analyze the pattern of pneumatization of the frontal recess by means of a tomographic study, to determine the prevalence of cell types in this region and their association with the development of rhinosinusitis.

## Methods

### Study procedures

We retrospectively evaluated 300 Cone Beam computed tomography scans of the paranasal sinuses of patients with clinical suspicion of chronic rhinosinusitis seen at Instituto Paranaense de Otorrinolaringologia Hospital between January 2014 and June 2019. The images were obtained using an iCat-1 Cone Beam CT scanner (Sciences International, Hatfield, USA) with a slice thickness of 0.25 mm. The data was analyzed by the author using Arya software, which allows simultaneous reconstruction of the three tomographic planes. Each side of the patient was studied separately, thus accounting for a final sample size of 600. On each side of the paranasal sinuses, the prevalence of cells present in the recess and frontal sinus region was assessed, as well as the presence of blockage and/or veiling in this anatomical region.

The classification proposed in 2016 by Wormald was used for this study, whereby the cells present in the region of the recess and frontal sinus could be classified as: supra-Agger cells, supra-Agger frontal cells, supra bulla cells, supra bulla frontal cells, supraorbital ethmoid cells and frontal septal cells. Other information obtained by analyzing the images was the presence of frontal sinusopathy, defined when there was a blockage and/or veiling in the region of the recess and frontal sinus. This information was then tabulated. Thus, after statistical evaluation, it was possible to compare whether the presence and number of frontoethmoidal cells were associated with the development of frontal sinusopathy.

### Statistical analysis

The data was analyzed using the computer program Stata/SE v.14.1. StataCorpLP, USA. The estimated measure of association was the Odds Ratio, for which 95% Confidence Intervals were presented. Values of p < 0.05 indicated statistical significance.

### Ethics committee approval

This study was approved by the Research Ethics Committee of Hospital de Clínicas da Universidade Federal do Paraná - HC/UFPR ‒ on June 6, 2019, under opinion number 3.373.545. This research was conducted in accordance with the principles of the Helsinki Declaration of 1975.

## Results

### Demographic data

The gender distribution was 179 female patients (59.7%) and 121 male patients (40.3%), with a mean age of 37.4-years, a minimum variation of 14-years and a maximum of 87-years.

### Prevalence of recess and frontal sinus cells

Frontoethmoidal cells were present in 85.8% of cases, with supra bulla cells being the most frequent in 43.8% of cases and supraorbital ethmoid cells the least frequent in 11% of cases. The general distribution of these cells was as follows: supra-Agger cells had a prevalence of 35%, supra-Agger frontal cells were present in 15.8% of cases, supra bulla cells were present in 43.8% of cases, supra bulla frontal cells had a frequency of 20.2%, frontal septal cells were frequent in 12.3% and supraorbital ethmoid cells had a prevalence of 11% and in 44.2% of cases there were two or more cells present in this region.

### Prevalence of the presence of blockage and/or veiling in the recess and frontal sinus region

The variables blockage and/or veiling of the recess and frontal sinus define the presence of sinusopathy in this paranasal sinus. The blockage variable was present in 30.3% of cases and the veiling variable was present in 11.7% of cases. The presence of blockage and/or veiling was prevalent in 30.7% of patients.

### Association between frontal recess cell types and the presence of frontal recess blockage

#### Univariate analysis

For each of the variables, the null hypothesis that there is no association between the variable and the probability of having a blockage was tested against the alternative hypothesis that there is an association.

The Table 1 shows the frequencies and percentages of cases with blockages according to the classifications of the variables, the p-values of the statistical tests and the estimated Odds Ratio (OR) values, with the respective 95% Confidence Intervals. The percentages of sinus with blockages were calculated in relation to the total of each classification of the variable analyzed, i.e., in relation to “n”.

**Table 1:** Relationship of frontoethomoid cells with frontal recess blocking.

Variable	Classification	N	Blocking. n (%)	*p*^a^	OR	95% CI
Supra-Agger cells	No	390	124 (31.8%)			
	Yes	210	58 (27.6%)	0.289	0.82	0.57‒1.19
Supra-Agger Frontal cells	No	505	144 (28.5%)			
	Yes	95	38 (40%)	0.026	1.67	1.06‒2.63
Supra Bulla Frontal cells	No	479	132 (27.6%)			
	Yes	121	50 (41.3%)	0.004	1.85	1.22‒2.80
Frontal Septal cells	No	526	162 (30.8%)			
	Yes	74	20 (27%)	0.509	0.83	0.48‒1.44
Supra Bulla cells	No	337	112 (33.2%)			
	Yes	263	70 (26.6%)	0.081	0.73	0.51‒1.04
Supraorbital Ethmoid cells	No	534	158 (29.6%)			
	Yes	66	24 (36.4%)	0.260	1.36	0.80‒2.32

a Logistic regression model and Wald test, *p* < 0.05.

The presence of two or more cells in the frontal region was not associated with a greater propensity to develop the blockade variant; however, when one of the cells presents was the supra-Agger frontal cell or the supra bulla frontal cell, the patient was positively associated with the blockage outcome (Table 2).

**Table 2:** Quantity of frontoetmoid cells × frontal recess blockage.

Variable	Classification	N	Blocking. n (%)	*p*^a^	OR	95% CI
2 or more cells	No	335	104 (31%)			
	Yes	265	78 (29.4%)	0.670	0.93	0.65‒1.32
2 or more (Supra-Agger Frontal cells)	No	529	153 (28.9%)			
	Yes	71	29 (40.9%)	0.042	1.70	1.02‒2.82
2 or more (Supra Bulla Frontal cells)	No	520	147 (28.3%)			
	Yes	80	35 (43.8%)	0.006	1.97	1.22‒3.19

a Logistic regression model and Wald test, *p* < 0.05.

After performing statistical tests, we observed that the presence of supra-Agger frontal cells and supra bulla frontal cells showed a significant correlation with the development of blockage in the frontal recess region.

#### Multivariate analysis

To jointly assess the association of variables with the probability of blockage, a multivariate Logistic Regression model was fitted including the variables that showed a *p-*value of < 0.10 in the univariate analysis. The results indicate that, regardless of the presence of supra-Agger frontal cells or supra bulla cells, the presence of supra bulla frontal cells is significantly associated with the likelihood of having a block (*p* = 0.032). A patient with supra bulla frontal cells is 64% more likely to have a blockage than a patient without a supra bulla frontal cell (Table 3).

**Table 3:** Multivaried analysis of frontoetmoid cells × blocking.

Variable	*p*^a^	OR	95% CI
Supra-Agger Frontal cells	0.079	1.51	0.95‒2.41
Supra Bulla Frontal cells	0.032	1.64	1.04‒2.58
Supra Bulla cells	0.542	0.89	0.60‒1.31

a Logistic regression model and Wald test, *p* < 0.05.

### Association between frontal recess cell types and the presence of frontal sinus veiling

For each of the variables, the null hypothesis that there is no association between the variable and the probability of having a veil was tested against the alternative hypothesis that there is an association.

The Table 4 shows the frequencies and percentages of cases with veiling according to the classifications of the variables, the p-values of the statistical tests and the estimated Odds Ratio (OR) values, with the respective 95% Confidence Intervals. The percentages of sinus with veiling were calculated in relation to the total of each classification of the variable analyzed, i.e., in relation to “n”.

**Table 4:** Relationship between frontoethmoidal cells and veiling of the frontal sinus.

Variable	Classification	N	Veiling. n (%)	*p*^a^	OR	95% CI
Supra-Agger cells	No	390	53 (13.6%)			
	Yes	210	17 (8.1%)	0.048	0.56	0.32–1.00
Supra-Agger Frontal cells	No	505	53 (10.5%)			
	Yes	95	17 (17.9%)	0.042	1.86	1.02‒3.38
Supra Bulla Frontal cells	No	479	36 (7.5%)			
	Yes	121	34 (28.1%)	<0.001	4.81	2.85‒8.11
Frontal Septal cells	No	526	60 (11.4%)			
	Yes	74	10 (13.5%)	0.598	1.21	0.59‒2.49
Supra Bulla cells	No	337	47 (14%)			
	Yes	263	23 (8.8%)	0.051	0.59	0.35–1.002
Supraorbital Ethmoid cells	No	534	61 (11.4%)			
	Yes	66	9 (13.6%)	0.598	1.22	0.58‒2.60

a Logistic regression model and Wald test, *p* < 0.05.

The presence of two or more cells in the frontal region was not correlated with the outcome of veiling; however, if one of the cells presents was a supra-Agger frontal cell or a supra bulla frontal cell, the patient was more likely to have frontal veiling (Table 5).

**Table 5:** Quantity of frontoethmoid cells × frontal sinus veiling.

Variable	Classification	N	Veiling, n (%)	*p*^a^	OR	95% CI
2 or more cells	No	335	38 (11.3%)			
	Yes	265	32 (12.1%)	0.781	1.07	0.65‒1.77
2 or more cells (Supra-Agger Frontal cells)	No	529	56 (10.6%)			
	Yes	71	14 (19.7%)	0.027	2.08	1.09‒3.96
2 or more cells (Supra-Bulla Frontal cells)	No	520	49 (9.4%)			
	Yes	80	21 (26.3%)	<0.001	3.42	1.92‒6.10

a Logistic regression model and Wald test, *p* < 0.05.

After statistical analysis, we observed that the presence of supra-Agger frontal and supra bulla frontal cells showed a statistically significant relationship with the development of frontal sinus veiling.

#### Multivariate analysis

The results indicate that the presence of a supra bulla frontal cell is significantly associated with the probability of having veiling (*p* < 0.001), regardless of the other variables included in the model (Table 6).

**Table 6:** Frontoetmoid cells multivaried analysis.

Variable	*p*^a^	OR	95% CI
Supra-Agger Cells	0.263	0.70	0.38‒1.31
Supra-Agger Frontal Cells	0.516	1.25	0.64‒2.42
Supra Bulla Frontal Cells	<0.001	4.47	2.60‒7.68
Supra Bulla cells	0.331	0.66	0.29‒1.52

a Logistic regression model and Wald test, *p* < 0.05.

## Discussion

The frontal recess region is an area of complex anatomy, shaped like an inverted funnel or cone with an apex at the frontal ostium, and can be pneumatized by various ethmoidal cells. This study aims to establish the relationship between frontoethmoidal cells and the development of frontal disease, since it is widely accepted that obstruction of the frontal recess, either due to anatomical variation and/or mucosal inflammation, is one of the pathophysiological mechanisms of rhinosinusitis.^8^

Initially assessed using coronal and axial sections, a perfect anatomical understanding of this region was difficult. The advent of multiplanar tomography using parasagittal reconstruction has significantly improved the understanding of this space. Information such as the size and diameter of the frontal recess, the thickness of the frontal beak and the three-dimensional reconstruction of the position of the ethmoidal cells can be obtained with this form of tomographic reconstruction.^1^

Kew et al. and Choby et al. argue that tomographic analysis in multiple planes improves the understanding of frontal anatomy in 95% of cases.^9^, ^10^ This multiplanar review also led to changes in surgical planning in 55% of cases when compared to uniplanar analysis.

The prevalence of frontoethmoidal cells, analyzed by means of CT scans, appears to vary in the studies. There are several classification systems for frontal cells and the vast majority of studies published to date use the old classifications proposed by Bent et al. in 1994. To date, only two studies have used the more modern classification, proposed by Wormald et al. in 2016, but none of these studies included the Brazilian population.^11^

This study found a prevalence of frontoethmoidal cells of 85.8%, which is higher than that found in previous studies. This finding may have been due to the combination of a multiplanar tomographic analysis and the use of the new classification “The International Frontal Sinus Anatomy Classification”, proposed in 2016.^12^ With the use of this classification, all the cells that can pneumatize the frontal recess region are analyzed together, namely: supra Agger cells, supra-Agger frontal cells, supra bulla cells, supra bulla frontal cells, supraorbital ethmoid cells and frontal septal cells, unlike what is observed in previous studies, when partial groups of cells are included, as can be seen in the studies by Meyer et al., DelGaudio et al. and Lee et al.^5^, ^7^, ^13^

Meyer et al. in 2003 described a prevalence of frontal cells of 20.4%.^5^ In this analysis, the classification proposed by Bent in 1994 was still used and only cells K1 to K4 were included. DelGaudio et al. found a frequency of 33% for frontal cells and also in this study only K1–K4 cells were considered.^7^ Van Alyea (1941) reported that frontal cells were present in 41% of cases.^14^ In this analysis, Agger nasi cell, supraorbital ethmoid cells and frontal septal cells were considered, in addition to K1–K4 cells.^15^

The present study may have shown a higher prevalence of frontoethmoidal cells due to the anatomical peculiarities of the Brazilian population, as seen by Kubota et al.,^8^ who studied the anatomy of the frontal recess in the Japanese and compared it with the pneumatization pattern of the Chinese, Koreans and Taiwanese. This finding was also present in the study by Cho et al.,^16^ when they compared the frontal pneumatization of Koreans and Caucasians and suggested a higher frequency of frontal cell type 1, frontal cell type 2 and supraorbital ethmoid cells in Caucasians because they have a more pronounced glabella and superior orbital rim than Koreans.

The Agger nasi cell is the most anteriorly pneumatized anterior ethmoidal cell, documented at a frequency of around 95.7%,^11^ but there are reports of a range from 89% to 98%. This highlights its constant presence and justifies it being used as an anatomical landmark for surgical access to the frontal region and also being the reference cell for the classification proposed in 2016 “The International Frontal Sinus Anatomy Classification”.^11^, ^17^

The pathophysiology of frontal sinusitis is associated with drainage and ventilation disorders of the frontal sinus through its ostium.^17^ The size of the frontal ostium is key to the drainage of this sinus. In 1991, Stammberger proposed the theory that stenosis of the ostiomeatal complex, whether due to anatomical configuration or mucosal hypertrophy, can cause obstruction and stagnation of secretions that can become infected or perpetuate infections. Thus, it is hypothesized that the presence of frontoethmoidal cells associated or not with their inflammation or infection can cause a narrowing of the frontal sinus drainage pathway and thus predispose to the development of rhinosinusitis.^8^

During endoscopic frontal sinusotomy, the complete removal of these cells is necessary to ensure adequate patency for drainage and ventilation of this sinus. Anatomical causes of persistent obstruction of the frontal recess have been described as one of the main causes of surgical failure.^4^ Van Alyea, in 1941, already warned about a series of cells that could be present in the frontal recess region and cause obstruction of its drainage, in addition to warning that inadequate removal of these cells could lead to the development of iatrogenic chronic frontal sinusites.^18^

This study analyzed the association between the presence of cells in the frontal recess and the development of sinusopathy in this sinus. Univariate analysis found a relationship between supra agger frontal cells and supra bulla frontal cells and the development of frontal sinus blockage and/or veiling. It was also assessed the number of cells present in the frontal recess region, when more than one, would correlate with sinusopathy. No statistical significance was found in this analysis; however, when there was more than one cell in the frontal recess region and one of them was the supra-Agger frontal cell or the supra bulla frontal cell, there was a statistically significant relationship with the development of frontal disease. Multivariate analysis was carried out for each variable and the cell type that remained independent for the development of sinusopathy was the supra bulla frontal cells.

These findings are in accordance with the data from the studies proposed by Meyer et al., Kubota et al. and Makihara et al.^5^, ^8^, ^19^ The study by Kubota et al. identified an association between the supra bulla frontal cells and the development of frontal sinusitis, since these cells are significantly associated with a narrowing of the anteroposterior diameter of the frontal recess^8^ Lien et al. reported in their study that the posterior frontoethmoidal cells show a greater association of statistical significance with the development of frontal sinusitis,^4^ which is in line with the multivariate analysis of the present study, which states that the posterior supra bulla frontal cells are an independent factor in the development of frontal rhinosinusitis.

## Conclusion

The presence of supra-Agger frontal and supra bulla frontal cells showed an association with the development of frontal sinusopathy. After multivariate analysis, the supra bulla frontal cell was the cell that remained an independent factor for the outcomes of blockage or veiling (frontal sinusopathy).

Successful surgical treatment of frontal rhinosinusitis depends on a three-dimensional anatomical understanding of the recess and frontal sinus region, which can be achieved through a preoperative tomographic study of the pneumatization pattern of this anatomical region. Through a rich knowledge of this anatomy, we can reduce surgical complications and the possible recurrence of the disease.

## Funding

There is no fundings.

## Declaration of competing interest

The authors declare no conflicts of interest.
